# A LASSO-based predictive nomogram for obstructive coronary artery disease in double zero score patients: validation and cardiovascular education strategies

**DOI:** 10.3389/fcvm.2025.1628622

**Published:** 2025-10-15

**Authors:** Chia-Hao Liang, Yi-Chi Hung, Hsin-Hung Chen, Yun-Ju Wu, Shu-Ching Yang, Wen-Hwa Wang, Fu-Zong Wu

**Affiliations:** ^1^ Ph.D. Program of Interdisciplinary Medicine, National Yang Ming Chiao Tung University, Taipei, Taiwan; ^2^ Department of Medical Education and Research, Kaohsiung Veterans General Hospital, Kaohsiung, Taiwan; ^3^ Department of Radiology, Kaohsiung Veterans General Hospital, Kaohsiung, Taiwan; ^4^ Laboratory of Tissue-Engineering, Department of Medical Imaging and Radiological Sciences, Central Taiwan University of Science and Technology, Taichung, Taiwan; ^5^ Department of Software Engineering and Management, National Kaohsiung Normal University, Kaohsiung, Taiwan; ^6^ Intelligent Electronic Commerce Research Center, Institute of Education, National Sun Yat-Sen University, Kaohsiung, Taiwan; ^7^ Department of Cardiology, Kaohsiung Veterans General Hospital, Kaohsiung, Taiwan; ^8^ Department of Post-Baccalaureate Medicine, National Sun Yat-Sen University, Kaohsiung, Taiwan; ^9^ Faculty of Medicine, School of Medicine, National Yang Ming Chiao Tung University, Taipei, Taiwan

**Keywords:** zero score, LASSO, coronary CTangiography, obstructive CAD, plaque

## Abstract

**Background:**

This study aimed to create a nomogram to predict a double-zero score for obstructive coronary artery disease (CAD) in a hospital-based cohort initially scoring zero. We compared its diagnostic performance with the Framingham risk score (FRS) and models for atherosclerotic cardiovascular disease (ASCVD).

**Methods:**

We retrospectively reviewed the clinical features and laboratory profiles of 634 participants with baseline zero coronary artery calcium scores. The target population consisted of individuals with a double-zero score. The primary endpoint was the diagnosis of obstructive CAD, defined as CAD-RADS ≥3 or vulnerable plaque formation on the second cardiac CT. The control group had a double-zero score, with no or less than 50% coronary stenosis. We developed a nomogram using a least absolute shrinkage and selection operation-derived logistic model. We assessed the models’ discrimination and calibration abilities using the Hosmer–Lemeshow test.

**Results:**

Participants were monitored for an average period of 4.26 ± 2.30 years and were randomly allocated to training and validation sets at a ratio of 2.8:1. The study results indicated that 5.13% (24 of 467) in the training cohort and 4.19% (7 of 167) in the validation cohort developed a double-zero score with obstructive CAD progression. This nomogram incorporated four predictors: “systolic blood pressure,” “hypertension,” “body fat percentage,” and “HbA1c.” The nomogram demonstrated superior diagnostic performance compared to the FRS and ASCVD models, with lower values of Akaike information criterion and Bayesian information criterion values. The nomogram's discriminative ability, measured by the area under the curve, was 0.792 in the training cohort and 0.824 in the validation cohort.

**Conclusions:**

The validated nomogram provides valuable predictive potential for identifying high-risk subclinical coronary atherosclerosis, thereby supporting personalized primary prevention and education strategies.

## 1 Introduction

In recent years, coronary computed tomography angiography (CTA) has been widely employed in the diagnostic evaluation of coronary artery disease (CAD) in intermediate-to-high-risk populations. Its use for the subclinical assessment of coronary atherosclerosis in low-to-intermediate-risk populations is also rising (1–5). As per a literature review, a calcium score of zero could serve as a gatekeeper to safely exclude the possibility of CAD in patients with low-to-intermediate risk, who also have a very low risk of cardiovascular events and mortality over a 10-year period (6–11). Recent studies have shown that while the mortality rate is low, nonfatal major adverse cardiovascular events (MACEs) are prevalent in low-to-intermediate-risk populations with zero calcium scores (5, 12, 13). Therefore, individuals with low-to-intermediate risk and zero calcium scores may exhibit early signs of CAD and bear a high risk of subclinical atherosclerosis (14–16).

Therefore, it is crucial to identify subclinical atherosclerosis, such as obstructive CAD or high-risk plaques, in patients with low-to-intermediate risk and double-zero scores during a series of CAC scans. These patients are potential candidates for medical therapy in preventive cardiology.

The effectiveness of the well-known Framingham risk score (FRS) or atherosclerotic cardiovascular disease (ASCVD) model in predicting the outcome of a double-zero score for obstructive CAD in patients with a baseline zero score remains uncertain. In a previous retrospective study, only moderate diagnostic performance of the FRS model was reported for the prediction of a double-zero score with obstructive CAD events (17). No previous studies have explored early preventive models to predict the risk of double-zero scores for obstructive CAD in Asian populations with a baseline zero score. With the widespread use of cardiac CT, certain populations may initially exhibit zero coronary scores in series scans, yet subsequently develop vulnerable soft plaques and obstructive coronary stenosis ≥50%. While existing literature shows that CAC = 0 does not rule out early CAD, the introduction could better emphasize the clinical significance, such as the risk of nonfatal cardiovascular events and missed chances for early preventive interventions.

As a double-zero score can be misinterpreted as low cardiovascular risk, it is crucial to identify this specific population in preventive medicine. Therefore, we aimed to develop and validate a least absolute shrinkage and selection operator (LASSO)-based risk model for predicting a double-zero score in obstructive CAD, with an initial score of zero, in the hospital-based cohort with low-to-intermediate risk. We also compared its discriminatory ability with the well-known FRS and ASCVD scores to determine its clinical utility.

## 2 Methods

### 2.1 Study design and population characteristics

**Figure 1:**
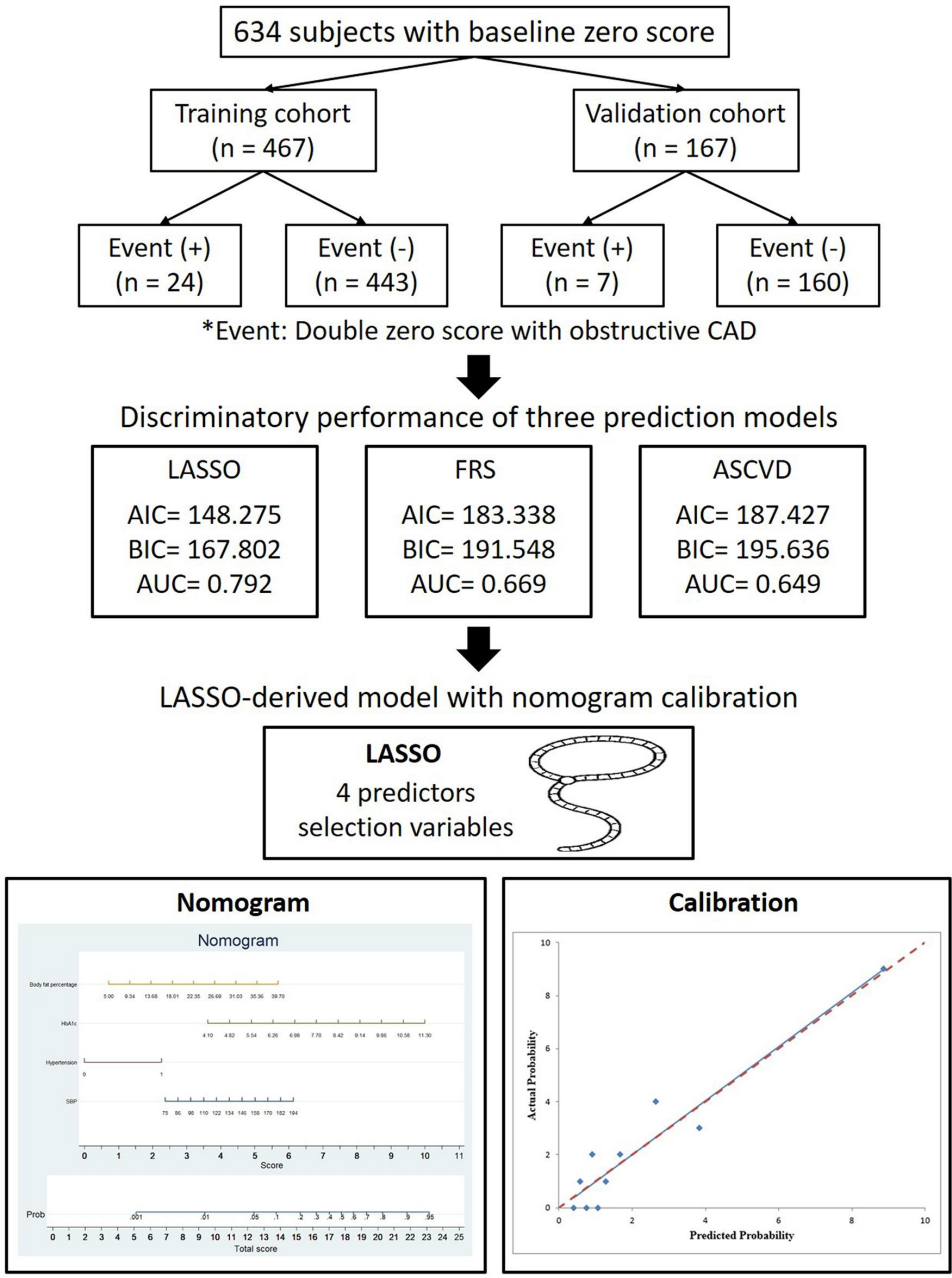
Flowchart depicting the selection process of patients for the training and validation cohorts in this study, focusing on the primary outcome of a double zero score with obstructive CAD.

A total of 634 consecutive patients from April 2005 to January 2022 were enrolled using the inclusion criteria mentioned in previous studies (17, 18). This study was designed as a retrospective cohort study conducted. Individuals with baseline zero calcium scores who underwent both baseline and follow-up CAC and coronary CT scans were included. At follow-up, participants were classified into two groups: those with obstructive CAD (≥50% stenosis or vulnerable plaque) and those without CAD. Inclusion criteria were individuals who underwent both baseline and follow-up CAC and coronary CT scans, with a baseline score of zero and a double-zero score on follow-up. Patients were categorized by the presence or absence of obstructive CAD (≥50% stenosis) or vulnerable plaques on follow-up scans. Exclusion criteria were those lacking the required scans or presenting non-zero baseline scores. Follow-up duration was not predefined and varied among participants. Repeat cardiac CT scans were performed based on clinical indications and physician judgment, without a standardized interval, reflecting real-world practice patterns in our setting. The prediction task was to develop a model identifying individuals at risk of developing obstructive CAD despite having a double-zero score. The primary endpoint was obstructive CAD, defined as ≥50% stenosis or the presence of vulnerable plaque, detected on the second cardiac CT scan. The time horizon for prediction extended from the baseline scan (with a zero calcium score) to the follow-up scan (double-zero score). All predictors, including demographic characteristics, clinical risk factors, and imaging features, were collected at baseline before the follow-up cardiac CT. As per the Declaration of Helsinki, the study protocol was approved by Kaohsiung Veterans General Hospital (IRB: VGHKS19-CT6–02, KSVGH21-CT7-22). The procedures undertaken in this study were conducted in compliance with institutional guidelines, and all methodologies adhered to the relevant regulations and guidelines. The requirement for patient consent was waived due to the retrospective study design. The primary endpoint of the study was to construct a LASSO-derived model to predict obstructive CAD with ≥50% stenosis with double-zero score events (defined as CAD-RADS ≥3 or vulnerable plaque formation in the second round of the CT exam, as described in our previous study) in the second round of the cardiac CT scan, and to assess its discriminatory power in comparison to other prediction models, such as the FRS and ASCVD score (17, 18). CAD-RADS ≥3 indicates significant coronary artery disease, denoting atherosclerotic plaque resulting in moderate stenosis or more, suggesting increased cardiovascular risks and necessitating precise management and treatment interventions. The control group had a double-zero score, with no coronary stenosis or stenosis <50%. We derived and internally validated the nomogram in 634 patients with a baseline zero score who were randomly assigned to the training (*N* = 467) and validation (*N* = 167) datasets at a ratio of 2.8:1. Figure 1 presents a flowchart of the study*.*

### 2.2 Data collection

Clinical and laboratory data were obtained from our hospital's electronic medical records using standardized forms. We included demographic variables such as age, sex, current smoking status, pack-year, hypertension, diabetes mellitus (DM), and follow-up period. Laboratory variables from blood, collected at the same time as the baseline scan, included uric acid, fasting glucose, C-reactive protein (CRP), gamma-glutamyl transferase (GGT), hemoglobin A1c (HbA1c), low-density lipoprotein cholesterol, total cholesterol, high-density lipoprotein cholesterol, and triglyceride levels. Additionally, a physical examination was conducted to collect anthropometric measurements of body mass index, body fat percentage, and waist circumference. Hypertension was defined as a systolic blood pressure (SBP) > 140 mmHg, diastolic blood pressure above 90 mmHg, or the use of hypertension medications.

DM was diagnosed in individuals receiving oral antidiabetic medications or insulin, which aligns with previous studies (17, 18).

### 2.3 Cardiac CT imaging acquisition and analysis

All patients underwent two consecutive scans employing a 256 × 0.625-mm detector row CT scanner, using either a 64 × 0.5-mm detector row CT vendor system (Aquilion 64; Toshiba Medical Systems) or a 256 × 0.625-mm detector row CT system (Revolution CT, GE Healthcare, Milwaukee, USA), as previously described.

The CT image acquisition protocol consisted of two sequential steps of imaging acquisition. The first step was a non-contrast coronary artery calcium (CAC) scan with the following acquisition settings: a fixed tube voltage of 120-kV and a 3 mm slice thickness reconstruction. The second step was a prospective ECG-triggered cardiac CTA with a fixed tube voltage of 120 kV and tube current modulation (mA modulation). CAC scores were calculated applying the Agatston method along with GE AW analysis software (version 3.2). The classification of coronary artery stenosis/vulnerable plaque characteristics with standardized reporting of individual patients was reported according to the Coronary Artery Disease Reporting and Data System (CAD-RADS™) (19).

### 2.4 Three prediction models for double-zero scores with obstructive CAD

We used a LASSO-derived regression model to build a new prediction model with an optimal lambda value that reduces the cross-validation error. Candidate predictors included demographics, risk factors, and imaging features; continuous variables retained linearity, missing data imputed, and LASSO used for variable selection. Four features were selected from the clinical and laboratory data in the training dataset: hypertension, SBP, HbA1c, and body fat percentage. We proposed a LASSO-derived model to predict the outcome of a double-zero score with obstructive CAD in patients with a baseline zero score and compared it to two well*-*known models for CAD risk prediction (FRS and ASCVD). The discriminative ability of the model fitness was tested for each model individually based on c-statistics, Akaike information criterion (AIC), and Bayesian information criterion (BIC) differences. A more discriminative model with better fitting performance was characterized by higher c-statistic values and lower AIC and BIC values, as per the interpretation of the study. The FRS was originally developed to assess an individual's 10-year risk likelihood of developing coronary heart disease. The FRS uses clinical and laboratory information on age, sex, total and HDL cholesterol levels, smoking status, systolic blood pressure, and DM (20).

The ASCVD risk model was originally designed to estimate the 10-year risk of the first fatal cardiovascular outcome in individuals aged 40–65 years. It uses clinical and laboratory information such as age, sex, race, smoking status, diastolic blood pressure, systolic blood pressure, and DM (21). Based on the estimated risk stratification in a 10-year ASCVD risk assessment, individuals are categorized as low-risk if <5%, borderline risk if 5%–7.5%, intermediate risk if 7.5%–20%, and high-risk if ≥20% (22).

### 2.5 Statistical analysis

Statistical analyses were conducted using Stata version 13.0 (Stata Corp., College Station, TX, USA) and SPSS for Windows (version 22.0; SPSS Inc., Chicago, IL, USA). Sample size was calculated for the primary endpoint assuming a 5% event rate, *α* = 0.05, 80% power, 10% attrition. The determined sample size was considered adequate to achieve the desired power and significance level given the assumed event rate and attrition. Variables with <5% missing values were analyzed using complete-case analysis, while those with 5%–20% missingness were addressed using multiple imputation. LASSO logistic regression was performed using the LASSOPACK package (including lasso2, cvlasso, and rlasso) in Stata 13.0 (23). The significance levels reported were two-tailed, and statistical significance was defined as *p* < 0.05. Missing data were handled using multiple imputations to ensure that the analysis included all available information without introducing bias. A fixed random seed was used to ensure reproducibility. Random numbers were generated for each patient using the runiform function, and patients were then allocated into validation (26.3%) and training (73.7%) sets using the split command based on the predefined 1:2.8 ratio. The clinical data of the study participants in the training and validation cohorts were compared using Student's t-test for continuous variables and the chi-square test for categorical variables. The primary goal of this study was to create a prediction nomogram using the LASSO-based methodology with optimal lambda selection, which could predict the risk of a double-zero score with obstructive CAD in participants with a baseline zero score (23). LASSO Logistic Regression was deemed the most appropriate method for balancing predictive accuracy and interpretability. The optimal penalty parameter (lambda) in the LASSO regression was selected using tenfold cross-validation via the lasso select command in Stata. The lambda value corresponding to the minimum cross-validation mean squared error (MSE) was chosen to ensure an optimal balance between model sparsity and predictive accuracy. The study used a multivariate logistic regression model involving multiple predictors to determine the 95% confidence interval (CI) and odds ratio (OR), which were subsequently used to create a nomogram based on the LASSO-derived parameters in the training cohort. Multicollinearity was evaluated using the variance inflation factor (VIF). All VIF values were lower than 10, indicating that multicollinearity is not a concern in this model. We evaluated and compared the discriminatory abilities of the three models using the C-statistic, BIC, and AIC (24). Receiver operating curve (ROC) analysis was conducted to evaluate the performance of the three predictive models. We compared the c-statistics of the three models using the method described by DeLong et al. (25).

The AUC, specificity, sensitivity, negative predictive value (NPV), positive predictive value (PPV), positive likelihood ratio (LR), and negative LR were calculated. The c-statistic ranged from 0.5, indicating no discriminatory ability, to 1.0, indicating complete discriminatory ability. Calibration was assessed using the Hosmer–Lemeshow test and by calibration plotting the predicted double-zero score with obstructive CAD against the actual rates in deciles of predicted risk (26). To assess model performance and ensure parsimony, we calculated the Akaike Information Criterion (AIC) and Bayesian Information Criterion (BIC) for model comparison and selection. Model discrimination was evaluated using the area under the receiver operating characteristic curve (AUC), with 95% confidence intervals derived from bootstrap resampling.

## 3 Results

In total, 634 eligible participants were included in the analysis and followed up for a mean duration of 4.26 ± 2.30 years. The prevalence of a double-zero score for obstructive CAD events in the study cohort was 5.13%. The participants were randomly allocated to either the training or validation datasets at a ratio of 2.8:1. Figure 1 illustrates that the study included 467 participants with zero baseline scores in the training cohort and 167 participants in the validation cohort. In the training dataset, 24/467 participants (5.13%) developed the events of double-zero score with obstructive CAD during the follow-up time of 4.31 ± 2.31 years, while in the validation dataset, 7/167 participants (4.19%) developed the events of double-zero score with obstructive CAD during the follow-up period of 4.12 ± 2.25 years. The basic clinical characteristics of the training cohort (467 participants, mean age 50.69 ± 8.35, 289/467 or 61.9% male) and the validation cohort (167 participants, mean age 50.68 ± 8.40, 109/167 or 65.3% male) are listed in Table 1. The participants in the training and validation datasets were similar in terms of clinical characteristics, physical examination results, and laboratory profiles.

**Table 1:** Characteristics of the clinical data in the training and validation sets.

Variable	Total patient cohort (*n* = 634)	Training set (*n* = 467)	Validation set (*n* = 167)	*P*-value
Age (years)	50.69 ± 8.36	50.69 ± 8.35	50.68 ± 8.40	0.990
Sex, *n* (%)				0.437
Male	398 (62.8%)	289 (61.9%)	109 (65.3%)	
Female	236 (37.2%)	178 (38.1%)	58 (34.7%)	
BMI (kg/m^2^)	24.48 ± 3.46	24.65 ± 3.48	24.01 ± 3.38	0.041
SBP (mmHg)	123.36 ± 16.95	123.54 ± 16.89	122.85 ± 17.16	0.660
DBP (mmHg)	77.66 ± 10.98	77.75 ± 10.92	77.42 ± 11.20	0.739
Hypertension, *n* (%)	171 (27.9%)	128 (28.4%)	43 (26.5%)	0.644
Smoking, *n* (%)	193 (32%)	137 (30.7%)	56 (35.4%)	0.274
DM, *n* (%)	75 (12.3%)	58 (12.9%)	17 (10.6%)	0.454
Uric acid (mg/dl)	6.20 ± 1.53	6.19 ± 1.51	6.21 ± 1.60	0.896
GGT (U/L)	38.10 ± 45.97	39.17 ± 51.12	35.21 ± 27.54	0.413
Fasting glucose (mg/dl)	100.17 ± 22.99	100.47 ± 23.15	99.30 ± 22.58	0.577
CRP (mg/dl)	0.19 ± 0.26	0.20 ± 0.26	0.18 ± 0.24	0.446
LDL-C (mg/dl)	115.21 ± 28.74	115.72 ± 28.81	113.79 ± 28.58	0.460
HDL-C (mg/dl)	48.00 ± 13.58	48.13 ± 13.74	47.64 ± 13.17	0.694
Cholesterol (mg/dl)	204.33 ± 37.03	205.88 ± 36.97	199.99 ± 36.98	0.081
Triglycerides (mg/dl)	149.03 ± 101.91	151.08 ± 101.05	143.29 ± 104.38	0.401
HbA1c (%)	5.86 ± 0.80	5.87 ± 0.84	5.82 ± 0.70	0.450
Body fat percentage (%)	24.02 ± 6.02	24.27 ± 5.91	23.35 ± 6.27	0.122
Waist circumference (cm)	84.90 ± 9.37	85.21 ± 9.37	84.08 ± 9.37	0.227
Follow-up period (year)	4.26 ± 2.30	4.31 ± 2.31	4.12 ± 2.25	0.377

BMI, body mass index; CRP, c-reactive protein; DM, diabetes mellitus; DBP, diastolic blood pressure; GGT, gamma-glutamyl transferase; HbA1c, hemoglobin A1c; HDL-C, high-density lipoprotein cholesterol; LDL-C, low-density lipoprotein cholesterol; SBP, systolic blood pressure.

Missing data: BMI, *n* = 4 (0.63%); SBP, *n* = 18 (2.84%); DBP, *n* = 18 (2.84%); Uric acid, *n* = 33 (5.21%); GGT, *n* = 174 (27.44%); Fasting glucose, *n* = 14 (2.21%); CRP, *n* = 34 (5.36%); LDL-C, *n* = 11 (1.74%); HDL-C, *n* = 11 (1.74%); Cholesterol, *n* = 11 (1.74%); Triglycerides, *n* = 11 (1.74%); HbA1c, *n* = 18 (2.84%); Body fat percentage, *n* = 120 (18.93%); Waist circumference, *n* = 126 (19.87%).

### 3.1 LASSO-derived predictive model for double zero score with obstructive CAD

To select the most important features and reduce dimensionality, we utilized LASSO regression with penalization and conducted a 10-fold cross-validation. We constructed a LASSO-derived prediction model, which selected four variables from the candidate variables in the training dataset for a double-zero score with obstructive CAD prediction in patients with low-to-intermediate risk. The final LASSO model with optimal lambda included four non-zero variables: hypertension, SBP, HbA1c, and body fat percentage. Table 2 summarizes the results obtained from the multivariate logistic regression analysis.

**Table 2:** Predicting double zero score in obstructive CAD events via LASSO-based logistic regression model.

Variable	Coefficient	OR	95% CI	*P*-value
SBP	0.033	1.034	1.010–1.057	0.004
Hypertension	1.526	4.601	1.959–10.809	<0.001
HbA1c	0.604	1.83	1.335–2.508	<0.001
Body fat percentage	−0.064	0.937	0.874–1.005	0.072
LASSO-derived model				
Prediction model	7.034	1,135.098	55.119–23,375.57	<0.001

CI, confidence interval; HbA1c, hemoglobin A1c; LASSO, least absolute shrinkage and selection operator; OR, odds ratio; SBP, systolic blood pressure.

### 3.2 Development and validation of LASSO-derived nomogram

**Figure 2:**
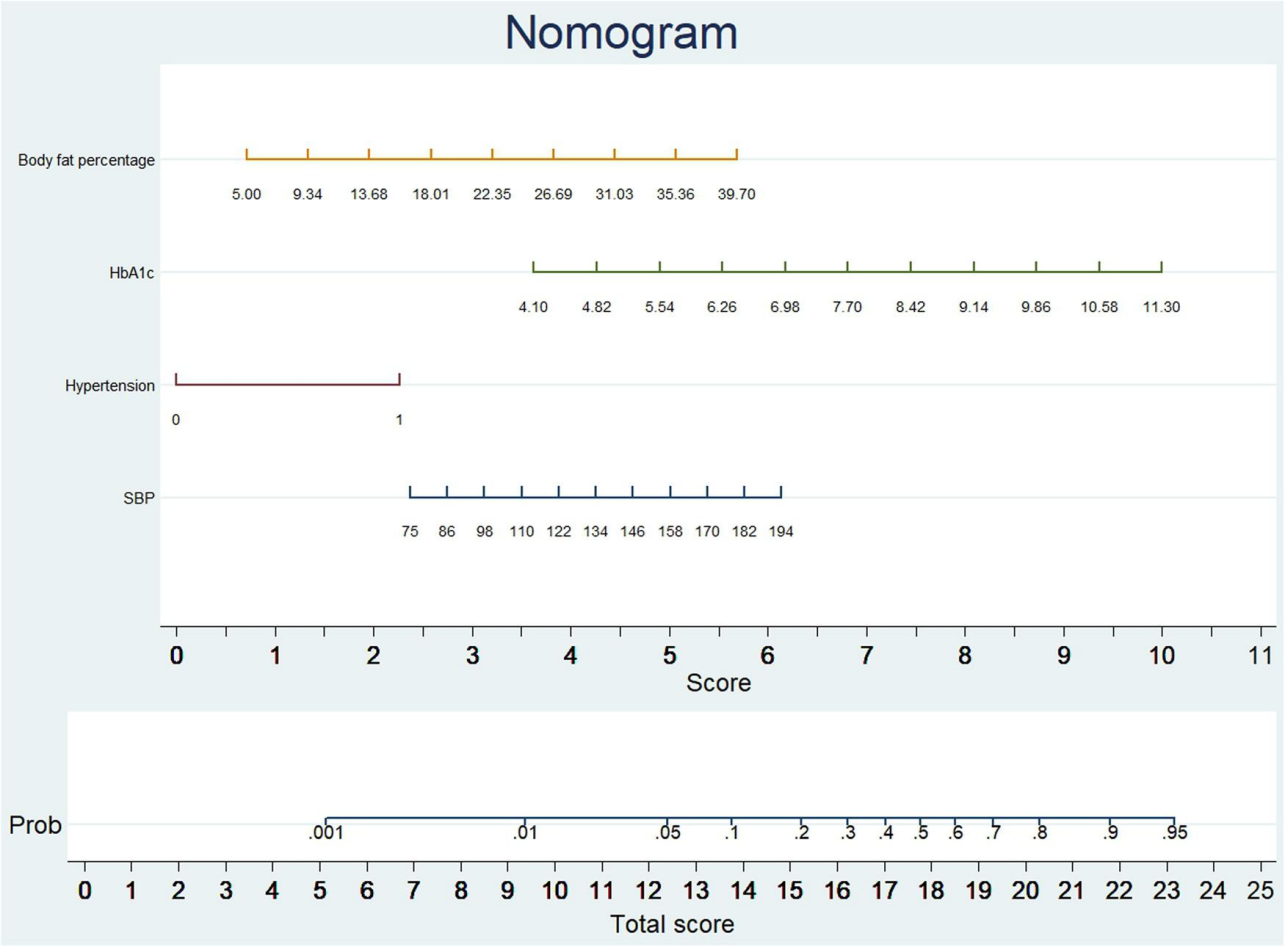
A nomogram was developed to predict obstructive CAD in individuals with a double-zero score. To use the nomogram, locate the value for each variable on its respective axis, and draw a line upwards to determine the corresponding number of points. The total number of points is then positioned on the total points axis to assess the likelihood of the primary outcome of a double-zero score with obstructive CAD.

**Figure 3:**
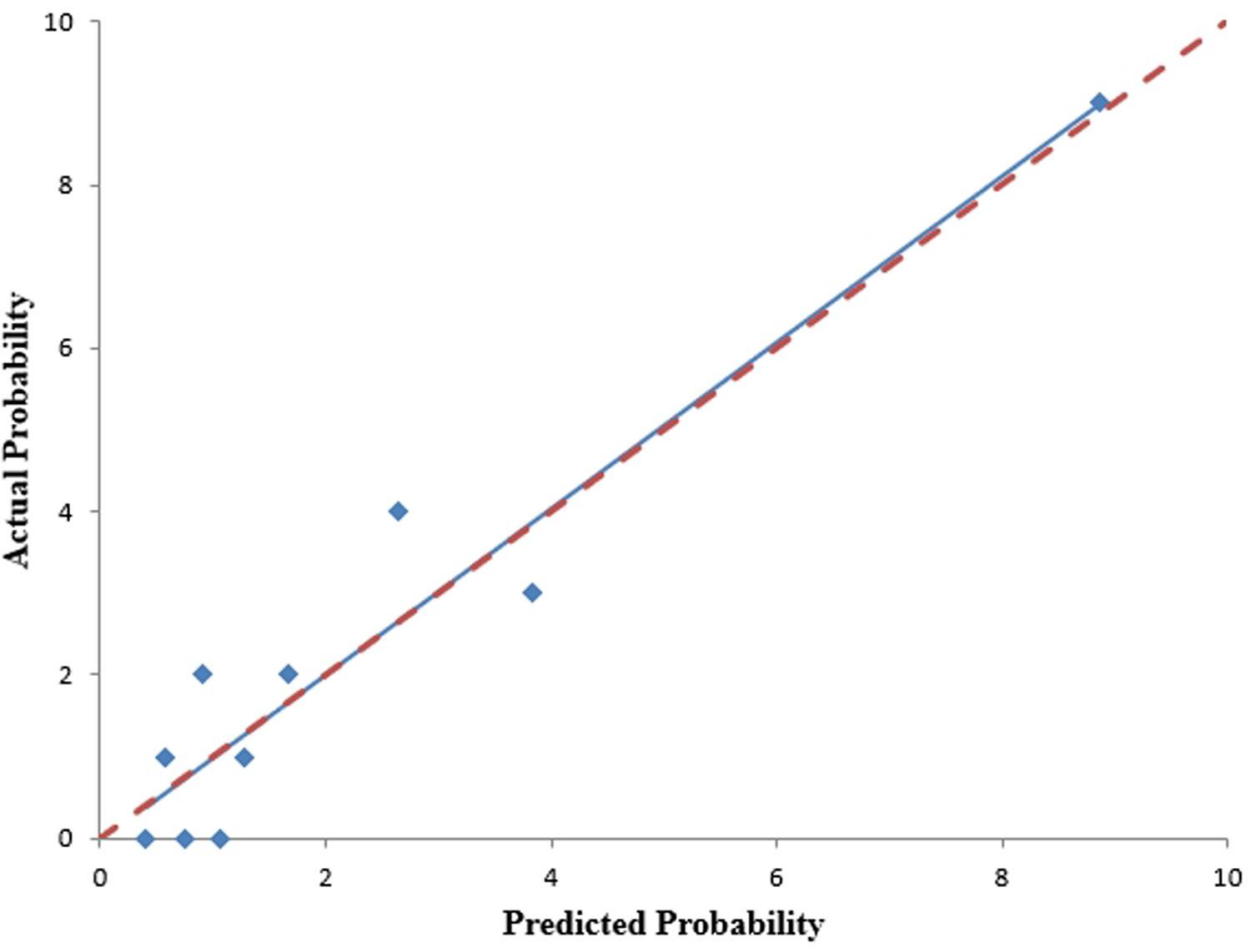
The nomogram's calibration curves, predicting the occurrence of a double-zero score with obstructive CAD, were generated using the training cohort. The Hosmer-Lemeshow test yielded a *p*-value of 0.732, indicating good calibration.

**Figure 4:**
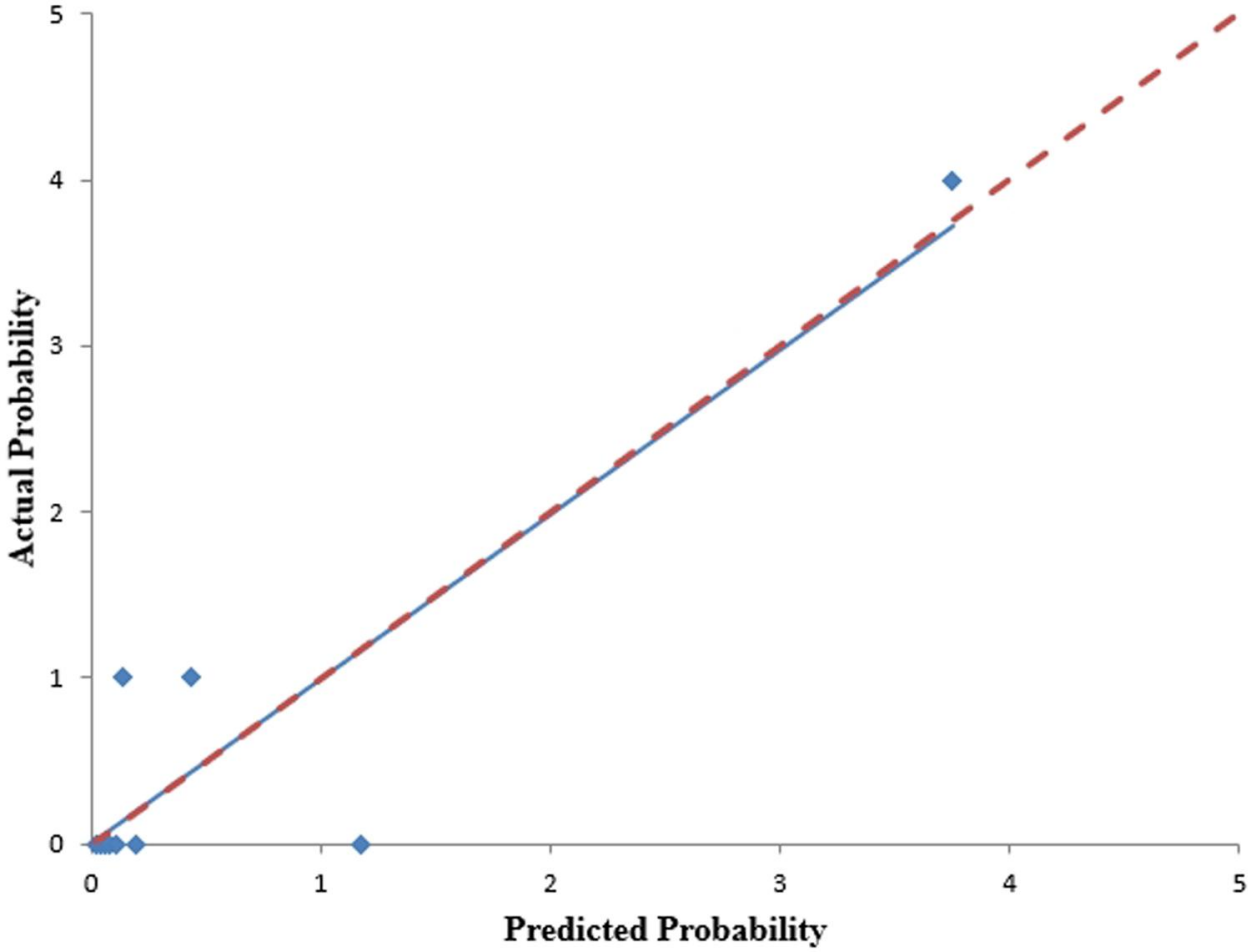
The calibration curves for the nomogram, predicting the occurrence of a double-zero score with obstructive CAD, were generated using the validation cohort. The Hosmer-Lemeshow test yielded a *p*-value of 0.966, indicating good calibration.

We analyzed the probability of a double-zero score for obstructive CAD in the training dataset with a baseline zero score using a multivariable logistic regression model that included four predictors: hypertension, SBP, HbA1c, and body fat percentage. A nomogram was generated to predict the double-zero score for obstructive CAD based on the multivariate logistic regression results shown in Figure 2. The nomogram showed favorable performance in predicting the double-zero score with obstructive CAD event, with an AUC of 0.792 (95% CI, 0.688–0.895) in the training dataset, which could also accurately classify participants into low-risk or high-risk subgroups. By summing the scores determined on the point scale for each selected predictor, we could calculate the estimated individual probability of a double-zero score with obstructive CAD using a straightforward line-drawing method in the training dataset with a baseline zero score. For instance, to provide a clearer explanation of the nomogram model, if a male patient with the selected predictor profile had hypertension (+), SBP = 158 mmHg, HbA1c = 11.30%, and body fat percentage = 35.36%, the probability of obstructive CAD with a double-zero score was estimated to be 93%. The Hosmer–Lemeshow test was applied to assess and validate the goodness-of-fit of the LASSO-derived prediction model/nomogram using the 10-fold cross-validation method. The pooled area under the ROC curve of the nomogram was 0.792 (95% CI: 0.688–0.895) in the training dataset and 0.824 (95% CI: 0.621–1.000) in the validation dataset. The ROC curve demonstrated that the resulting model had a favorable discrimination ability for both the training and validation datasets. The Hosmer–Lemeshow test yielded *p*-values of 0.732 and 0.966 in the training and validation datasets, respectively, indicating strong calibration performance, as illustrated by the calibration curves in Figures 3, 4.

### 3.3 Comparison of LASSO-derived, FRS, and ASCVD models

We assessed the diagnostic performances of the three predictive models using ROC curve analysis (Table 3). In addition, the comparison and model fitting of the three predictive models are depicted based on the C-statistics, AIC, and BIC model selection criteria in Table 4. The results of our study indicated that the LASSO-based model outperformed the other two predictive models, with significantly higher c-statistics, better discriminatory ability, and lower AIC and BIC values. Compared with the FRS and ASCVD models, the LASSO-derived nomogram model showed significantly superior diagnostic performance with an AUC of 0.792 (95% CI, 0.688–0.895) for the detection of a double-zero score with obstructive CAD in an Asian population with a baseline zero score, with high sensitivity (81.82%) and low specificity (69.28%) (LASSO model vs. FRS model: AUC: 0.792 vs. 0.669, *p* = 0.030; LASSO model vs. ASCVD model: 0.792 vs. 0.649, *p* = 0.036).

**Table 3:** Comparison of prediction performance among the LASSO, FRS, and ASCVD models., *n* = 467.

Models	AIC	BIC	AUC (95% CI)	Cut-point	Sensitivity	95% CI	Specificity	95% CI	+LR	95% CI	−LR	95% CI	+PV	95% CI	−PV	95% CI
LASSO model	148.2756	167.8024	0.792 (0.688–0.895)	>0.0447	81.82	59.7–94.8	69.28	64.1–74.1	2.66	2.2–3.3	0.26	0.1–0.6	14.5	8.8–22.0	98.4	95.8–99.5
FRS model	183.3386	191.5482	0.669 (0.557–0.780)	>16.9	54.17	32.8–74.4	76.18	71.8–80.2	2.27	1.6–3.3	0.6	0.4–1.0	11.4	6.2–18.7	96.7	94.2–98.3
ASCVD model	187.4271	195.6367	0.649 (0.532–0.765)	>8	54.17	32.8–74.4	72.88	68.4–77.1	2	1.4–2.9	0.63	0.4–1.0	10.2	5.5–16.7	96.6	93.9–98.3

AIC, Akaike information criterion; ASCVD, atherosclerotic cardiovascular disease; AUC, area under the ROC curve; BIC, Bayesian information criterion; CI, confidence interval; FRS, Framingham risk score; LASSO, least absolute shrinkage and selection operator; LR, likelihood ratio; PV, predictive value.

**Table 4:** Comparing the ability to discriminate among three predictive models.

Model comparison	Difference between areas	SE	95% CI	*P*-value
LASSO vs. FRS	0.104	0.057	0.008–0.215	0.030
LASSO vs. ASCVD	0.13	0.0622	0.008–0.252	0.036
FRS vs. ASCVD	0.0267	0.0151	−0.002 to 0.056	0.076

ASCVD, atherosclerotic cardiovascular disease; CI, confidence interval; FRS, Framingham risk score; LASSO, least absolute shrinkage and selection operator; SE, standard error.

In addition, the LASSO-derived model had smaller AIC and BIC than the other FRS and ASCVD models, suggesting superior goodness*-*of*-*fit among the three predictive models.

## 4 Discussion

This study constructed and validated a new prediction model for a double-zero score with obstructive CAD prediction in patients with baseline zero scores, using four easily available clinical variables. The nomogram showed sufficient diagnostic accuracy and discrimination. This retrospective study is the first, to the best of our knowledge, to build a LASSO-derived prediction model to investigate the natural course of the subclinical coronary atherosclerosis burden in an asymptomatic Asian individuals with a baseline CAC score of zero in terms of a double-zero score with obstructive CAD. This study had three main findings. First, we developed a LASSO-derived novel nomogram prediction model based on four easily available predictors (hypertension, SBP, HbA1c, and body fat percentage) to predict double-zero scores with obstructive CAD events. We demonstrated that it provides a favorable diagnostic performance for predicting these events in an Asian cohort. Adopting the LASSO model facilitates the selection of pertinent clinical risk factors, therefore enabling the more efficient establishment of a new model for predicting double-zero scores in obstructive CAD. The effective predictive capabilities of HbA1c and body fat percentage in specific populations with coronary artery stenosis can aid the precise characterization of high-risk features within this specific group. Second, compared with the FRS and ASCVD models, the LASSO-derived model showed significantly better discriminatory ability and lower AIC and BIC. Third, the risk prediction model derived from the LASSO showed good calibration performance in both the training and validation cohorts. Finally, for a double-zero score with obstructive CAD prediction, the LASSO-derived model with an optimum cutoff value of <0.0447 (probability) may help confirm patients at low risk for ruling out these clinical scenarios and discriminate high-risk from low-risk patients in the low-to-intermediate-risk population (sensitivity, 81.82%; specificity, 69.28%).

This LASSO-derived model demonstrates low PPV and high NPV, highlighting its strong ability to rule out obstructive CAD safely and its value in guiding risk stratification among low-to-intermediate-risk patients.

Recent studies have shown a high variation rate of 7%–32% in significant CAD in patients with zero CAC scores (15, 16, 27–29). According to previous studies, a calcium score of zero is associated with a high negative predictive value for obstructive CAD, particularly in low-to-intermediate-risk populations, although its rule-out capability remains limited. However, the prognostic outcome of obstructive CAD in patients with a zero CAC score is relatively good (7–10, 14–16).

Previous studies have reported a good prognosis with a very low cardiac event rate (<1%) in an asymptomatic population with a zero score (6, 10, 30, 31). However, it is important to note that low-to-intermediate risk symptomatic/asymptomatic patients with a zero score may have early signs of CAD or vulnerable soft plaque formation. This carries a high risk of subclinical atherosclerosis and should not be “downgraded” and stratified as patients with true zero or double-zero scores without subclinical coronary atherosclerosis (5, 17, 32). To highlight the increased cardiovascular risk associated with CAD-RADS 3 with a double-zero score, vigilant monitoring and tailored management strategies are required to mitigate disease progression and potential adverse events. Therefore, developing an accurate prediction model to identify high-risk patients with double-zero scores and obstructive CAD is important.

For double-zero scores with obstructive CAD prediction, the LASSO-derived model with an optimal cutoff value of <0.0447 (probability score) may be an ideal screening tool, offering a high negative predictive value (NPV = 98.4%) to help rule out these clinical scenarios in the middle-aged Asian population with low to intermediate risk (sensitivity, 81.82%; specificity, 69.28%). Therefore, our proposed LASSO-derived model/nomogram may help confirm patients at low risk of double-zero scores with obstructive coronary artery atherosclerosis to enhance cardiovascular risk stratification and reclassification. The nomogram can guide clinical decision-making by identifying patients at higher risk, informing the need for additional imaging, preventive interventions, or lifestyle modifications, and supporting shared decision-making between clinicians and patients, thereby enhancing personalized risk stratification and management in preventive subclinical period. Our previous study showed that the FRS model has a poor to fair discrimination performance for individuals with a double-zero score for obstructive CAD (17). In this study, the LASSO-derived model demonstrated significantly superior performance, with an AUC of 0.792 (95% CI, 0.688–0.895) for the detection of subclinical atherosclerosis progression in terms of obstructive CAD, with a double-zero score in the Asian population, compared with the FRS and ASCVD models. The findings of this study are consistent with those of previous studies regarding the relatively low rate of early CAD findings demonstrated on cardiac CTA in populations with zero or double-zero scores (7, 9, 31). In addition, our LASSO-derived model was feasible for predicting obstructive CAD with double-zero score events and had high sensitivity to rule out this clinical scenario in the study cohort. Therefore, early identification of high-risk subgroups and detection of coronary atherosclerosis progression in its subclinical stage using cardiac CT could affect the prevention of cardiovascular events at an early stage and guide ASCVD primary prevention and management through health promotion/education strategies such as lifestyle changes, diet control, exercise plans, or medication adherence (33–35). In addition, a shared decision-making plan using this novel risk stratification nomogram for subclinical atherosclerosis prevention could guide personalized medicine management toward holistic medicine.

## 5 Limitations

Our study has some limitations. First, this retrospective study was based in one hospital with a self-referral Asian population at low to intermediate risk. Although this study also demonstrated a low rate of early CAD signs in a population with a double-zero score, the findings showed that conventional cardiovascular models, such as the FRS or ASCVD models, could not correctly predict and identify high-risk candidates with a double-zero score. Moreover, the generalizability of the prediction model results to Western populations is limited because this study was conducted in an Asian cohort. The model was internally validated only. Future work should include external validation in diverse, unselected populations to confirm generalizability and enhance real-world clinical applicability. Second, we did not investigate cardiovascular events or mortality in the primary endpoint analysis. However, our study design primarily aimed to identify high-risk candidates for subclinical atherosclerosis based on a double-zero score for obstructive CAD. This LASSO-derived model is a useful screening tool for excluding mostly low-risk patients with a double-zero score, and further, cardiac CTA is warranted for high-risk candidates to confirm the diagnosis. Third, we investigated a distinct type of subclinical coronary atherosclerosis associated with high-risk soft plaques and obstructive CAD. Therefore, this study did not investigate other types of subclinical atherosclerosis, such as mixed or calcified plaques. The study population was highly selected, including only individuals undergoing serial CAC and CTA scans. This introduces a selection bias and limits the generalizability of the findings. Further studies are needed to elucidate other forms of subclinical atherosclerosis that may help prevent specific plaque progression. Specifically, we recognize the need for further research to integrate the model's predictions with tailored intervention strategies, including optimizing medication selection and dosage adjustments for high-risk patients, as well as refining follow-up schedules for low-risk patients. Future studies should incorporate intervention trials to evaluate the effectiveness of these strategies and provide more actionable recommendations for clinical practice. Third, future studies should incorporate cross-validation and larger datasets to improve model reliability, minimize bias, and ensure robust generalizability across diverse populations, mitigating small sample size limitations. Forth, a high false positive rate (30.72%) may raise concerns, but since this study aims to identify subclinical high-risk groups without leading to harmful interventions, its impact on practical application is minimal. The focus remains on risk stratification rather than immediate treatment.

## 6 Conclusion

To summarize, a prediction model based on four simple clinical parameters was successfully developed and validated using LASSO, conveniently including “hypertension,” “SBP,” “HbA1c,” and “body fat percentage,” and demonstrated that it provides a favorable level of performance for predicting double-zero score with obstructive CAD in patients with a baseline zero score. This nomogram could assist clinicians in identifying high-risk subclinical coronary atherosclerosis subtypes in low-to-intermediate-risk populations and provide a valuable tool for personalized primary prevention in preventive cardiology.

## Data Availability

The raw data supporting the conclusions of this article will be made available by the authors, without undue reservation.
