# Supplementary figures and images for: A LASSO-based predictive nomogram for obstructive coronary artery disease in double zero score patients: validation and cardiovascular education strategies
Source: Front Cardiovasc Med. 2025 Oct 15;12:1628622. doi: 10.3389/fcvm.2025.1628622 (PMC12570095; doi:10.3389/fcvm.2025.1628622)

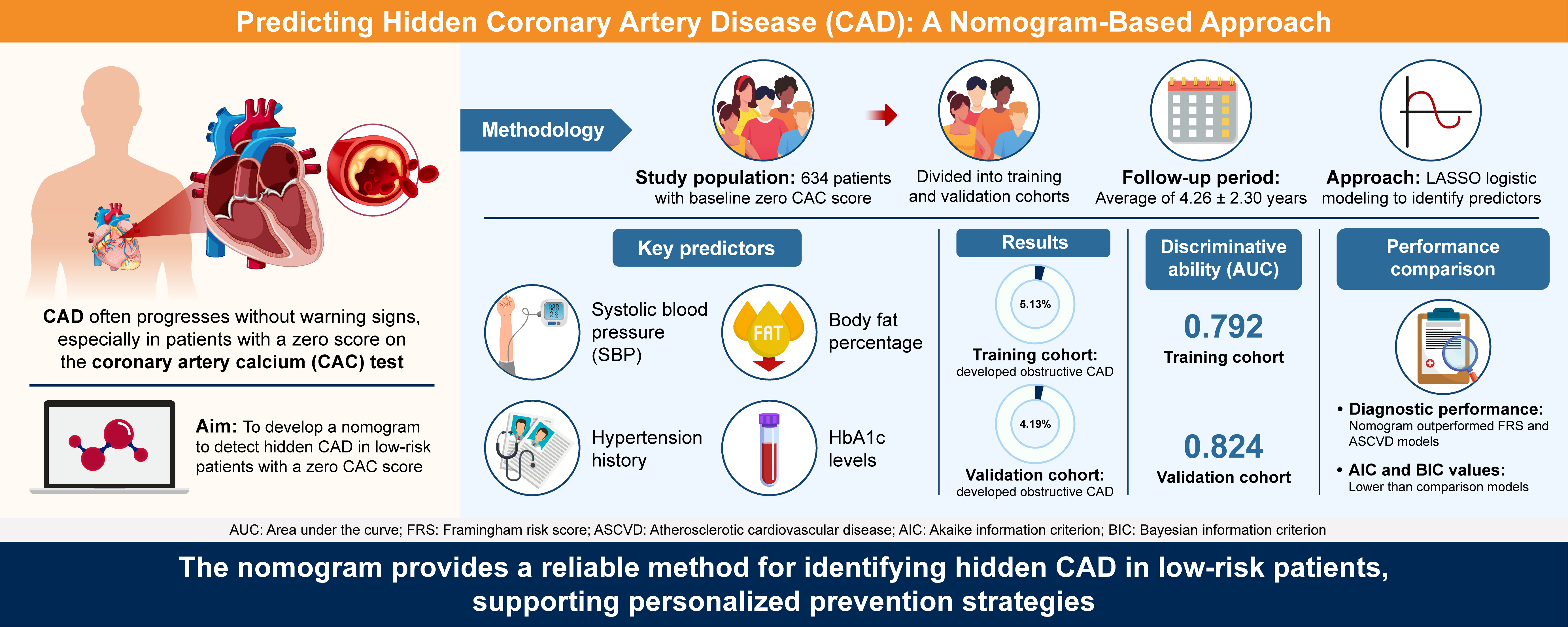

Supplement: Supplementary file 1 [file Image1.tif]
